# HbA1c: an independent risk factor for dysthyroid optic neuropathy

**DOI:** 10.3389/fendo.2023.1251209

**Published:** 2023-10-10

**Authors:** Xing Wang, Huijing Ye, Rongxin Chen, Shenglan Yang, Te Zhang, Wei Xiao, Huasheng Yang

**Affiliations:** ^1^ State Key Laboratory of Ophthalmology, Zhongshan Ophthalmic Center, Sun Yat-sen University, Guangdong Provincial Key Laboratory of Ophthalmology and Visual Science, Guangzhou, China; ^2^ Department of Ophthalmology, The First Affiliated Hospital of Chongqing Medical University, Chongqing Key Laboratory of Ophthalmology, Chongqing Eye Institute, Chongqing, China

**Keywords:** dysthyroid optic neuropathy, thyroid-associated ophthalmopathy, diabetes mellitus, HbA1c, risk factor

## Abstract

**Background:**

We aimed to explore the frequencies of islet β-cell autoantibodies and insulin resistance (IR) in thyroid-associated ophthalmopathy (TAO) and identify specific diabetes mellitus (DM) indicators as early predictors for dysthyroid optic neuropathy (DON).

**Methods:**

Ninety-eight TAO patients (57 DON and 41 non-DON patients) and 48 healthy control (HC) participants were recruited for this prospective cross-sectional study. Serum thyroxine, serum thyroid autoantibodies, serum humoral immune markers against islet β-cell, fasting plasma glucose (FPG), fasting serum insulin (FINS), fasting c-peptide (FCP), and glycosylated hemoglobin A1 (HbA1c) were measured. Logistic regression analysis was used to evaluate the correlation of patients’ age, body mass index (BMI), FPG, HbA1c, and related indexes of islet β-cell function to the occurrence of DON.

**Results:**

The DON group had higher FPG (P<0.001, 0.016) and HbA1c (P<0.0001, P<0.001) levels than the HC and non-DON groups. The homeostasis model assessment (HOMA)-IR level was the highest in the DON group (HC 2.15 ± 0.89, non-DON 2.41 ± 1.24, and DON 2.82 ± 2.65), while the HOMA-β level was the lowest (HC 101.8 ± 44.75%, non-DON 102.9 ± 54.61%, and DON 88.29 ± 52.75%), with no significant differences (P=1, P>0.05). On univariate analysis, age (P=0.006), BMI (P=0.022), history of steroid use (P=0.014), FPG (P=0.013), and HbA1c (P=0.001) levels were significantly associated with the presence/absence of DON. In addition, after adjusting for potential confounds, the HbA1c level was an independent factor associated with DON (P=0.009, OR=4.012).

**Conclusions:**

HbA1c is an independent risk factor for DON. Given the interconnected link between thyroid dysfunction and DM, the use of HbA1c as a potential biomarker for DON warrants further investigation.

## Highlights

**What is known?**

- DM and AITD are interconnected autoimmune endocrine diseases. The prevalence of combined thyroid dysfunction in patients with T2DM was reported to be 14.0% to 16.0%.
- Islet β-cell autoantibodies and function may play roles in thyroid dysfunction and TAO.

**What is new?**

- Dysthyroid optic neuropathy (DON) patients might have higher HbA1c and fasting plasma glucose.
- HbA1c is an independent risk factor for DON in thyroid-associated ophthalmopathy.
- Given the inextricable link between thyroid dysfunction and diabetes mellitus, the use of HbA1c as a potential biomarker for DON warrants further investigation.

## Introduction

Thyroid-associated ophthalmopathy (TAO) is one of the most common orbital diseases closely related to thyroid dysfunction in adults (1). It presents a spectrum of clinical manifestations, such as exophthalmos, diplopia, high orbital pressure, vision loss, and even blindness (2, 3). Severe forms of the disease affect approximately 3~5% of TAO patients, accompanied by severe eye pain, inflammation, corneal ulceration, or dysthyroid optic neuropathy (DON) (4). This vexing and undertreated ocular disease dramatically affects quality of life.

TAO severity is the result of complex interactions of thyroid dysfunction (5). Thyroid dysfunction is a systemic endocrine disease that includes hyperthyroidism, hypothyroidism, and autoimmune thyroid dysfunction (AITD). AITD comprises two main clinical presentations: Graves’ disease and Hashimoto’s thyroiditis. Graves’ disease is characterized by an overactive thyroid gland, leading to increased production of thyroid hormones. On the other hand, Hashimoto’s thyroiditis causes an underactive thyroid gland, leading to decreased production of thyroid hormones. Large fluctuations in thyroid stimulating hormone (TSH) and thyrotropin receptor antibody (TRAb) levels indicate a high risk of inducing severe exophthalmos (6, 7).

Diabetes mellitus (DM) is a group of metabolic diseases characterized by hyperglycemia. It is mainly divided into type 1 diabetes mellitus (T1DM, immune-mediated diabetes, and islet β-cell destruction leading to absolute insulin deficiency, with positive autoantibodies) and type 2 diabetes mellitus (T2DM, ranging from predominantly insulin resistance (IR) with relative insulin deficiency to predominantly an insulin secretory defect with IR). The global DM prevalence was 9.3% (463 million people) in 2019, which is projected to increase to 10.2% (578 million) by 2030 and 10.9% (700 million) by 2045 (8). More than 110 million people are presently estimated to be affected by DM in China, which has the highest number of people with DM globally (9).

Increasing evidence has shown that DM and AITD are interconnected autoimmune endocrine diseases (10–12). AITD and T1DM are organ-specific T-cell-mediated diseases. The combination of the two is called the autoimmune polyglandular syndrome type 3 variant, which is a complex multifactorial syndrome (13). It has been shown that patients with thyroid dysfunction have a higher chance of developing DM and vice versa (11, 14–17). Approximately 17~30% of T1DM adults experience AITD (10). Serum islet β-cell autoantibodies are the main humoral immune markers of T1DM, including insulin autoantibodies (IAA), islet cell autoantibodies (ICA), glutamic acid decarboxylase antibodies (GADA), tyrosine phosphatase antibodies (IA-2A), and zinc transporter 8 (ZnT8A) (18, 19). Previous studies found that there was a prevalence of GADA and ICA antibodies in the serum of AITD patients (20, 21). A total of 600 Caucasian patients with recently diagnosed Graves’ disease were characterized by a high prevalence of GADA antibodies (22). It implies that islet β-cell autoantibodies may also play a role in TAO.

In addition, the prevalence of combined thyroid dysfunction in patients with T2DM was reported to be 14.0% to 16.0% (23, 24). Patients with Graves’ disease developed thyroid dysfunction and DM earlier than patients with primary hypothyroidism (25). The basal plasma glucose concentration in patients with Graves’ disease was significantly higher than that in normal subjects (26). DM and smoking are major independent risk factors predictive of DON (27). T2DM with Graves’ disease has more frequent and severe TAO (28, 29). IR status is a continuous non-specific inflammatory process, with increased levels of the inflammatory cytokines interleukin (IL)-6, IL-1β, IL22, and tumor necrosis factor-α (30). Meanwhile, these inflammatory cytokines are also involved in pathogenesis in TAO (31, 32). Smoking, obesity, and a lack of Adenosine 5’-monophosphate-activated protein kinase in adipocytes can aggravate IR and glucose tolerance (33). The crosstalk between the insulin-like growth factor-1 (IGF-1) receptor and the TSH receptor plays a vital role in the pathogenesis of Graves’ disease and TAO (34, 35). IGFs may be biomarkers of pre-T1DM (36), and IGF-1 signaling is closely related to IR and DM (37). TSH, free thyroxine (FT4), free triiodothyronine (FT3), and dyslipidemia were significantly correlated with hyperglycemia and IR (38). IR and TAO are both non-specific inflammatory reactions with common roles in pathogenesis. Whether they crosstalk and influence each other has not been elucidated.

Among them, islet β-cell autoantibodies and function may play a role in thyroid dysfunction and TAO (39–41). Although several studies (27–29, 42) have reported an association between TAO and DM, the specific DM indicators that can be used as early biomarkers of DON remain unclear. The aim of this study was to investigate the frequencies of islet β-cell autoantibodies and IR in TAO in order to characterize the specific features of this group of endocrine patients and find risk factors for DON.

## Materials and methods

### Study participants

This was a prospective cross-sectional study conducted between August 2020 and June 2021 in the Department of Orbital Diseases of Zhongshan Ophthalmic Centre. This study was conducted following the Declaration of Helsinki, and written informed consent was obtained before any examination. Age- (≥ 18 years) and sex-matched TAO (DON group and non-DON group) patients and healthy control subjects (HC group) were enrolled simultaneously. Exclusion criteria for the participants were as follows: any ocular disease, any autoimmune-related disease, and pregnant and breastfeeding women. Written informed consent was obtained prior to any examination. Our study was conducted according to the Declaration of Helsinki and approved by the Institutional Review Board of Zhongshan Ophthalmic Center (2016KYPJ028).

### TAO evaluation

Bartley’s diagnostic criteria for Graves’ orbitopathy were used to diagnose TAO (43). The activity and severity of TAO were evaluated in line with the EUGOGO Guidelines (44). TAO activity was measured by the clinical activity score (CAS). This score is based on whether the following seven items are present: spontaneous retrobulbar pain, pain on attempted upward or downward gaze, redness of eyelids, redness of the conjunctiva, swelling of caruncle or plica, swelling of eyelids, and swelling of the conjunctiva. A CAS ≥ 3/7 indicates active TAO. The severity was graded according to the NOSPECS classification (0= no symptoms or signs; I= only signs, no symptoms; II= soft tissue involvement; III= proptosis; IV= extraocular muscle involvement; V= corneal involvement; VI= sight loss, due to optic nerve involvement). DON was diagnosed based on a combination of clinical and radiological findings (45): 1) decreased best-corrected visual acuity (BCVA) ≥ 2 lines on a logMAR visual chart, relative afferent pupillary defect (RAPD) when unilaterally affected, impairment of color vision, visual field (VF) defect (mean deviation in Humphrey perimetry < −10 dB), abnormal visual-evoked potential (VEP) test (latency delay and amplitude reduction), and apical orbital crowding in computed tomography (CT) or magnetic resonance imaging (MRI).

### Ophthalmic and systemic examinations

All subjects underwent comprehensive ocular examinations, including BCVA, Goldmann applanation tonometry, slit-lamp biomicroscopy, RAPD test, fundoscopy, and eye movement evaluation. For all TAO patients, the following ancillary tests were performed: color vision, Hertel exophthalmos, CAS score, severity, VF (Humphrey Field Analyser II 750; Carl Zeiss Meditec, Inc., Dublin, CA, USA), VEP (ESPION; Diagnosys LLC, Inc., Cambridge, UK), orbital CT (Mx8000 IDT; Philips, Amsterdam, The Netherlands), and/or MRI (Achieva 1.5T; Philips). Details of patients’ DM history, thyroid dysfunction history, TAO history, medical history, glucocorticoid therapy history, smoking history, height, and weight were collected before the examination.

### Laboratory measurements

The participants fasted for 8-12 hours before having venous blood collected from the cubital vein the following morning (8-9 a.m.). Serum levels of total triiodothyronine (TT3), total thyroxine (TT4), FT3, FT4, TSH, TRAb, thyroid peroxidase antibody (TPOAb), and antithyroglobulin antibody (TgAb) were measured. Primary serum humoral immune markers against islet β-cell were measured, including IAA, ICA, GADA, IA-2A, and ZnT8A. Furthermore, fasting plasma glucose (FPG), fasting serum insulin (FINS), fasting c-peptide (FCP), glycosylated hemoglobin A1 (HbA1c), total serum cholesterol, and triglycerides were also recorded. Homeostasis model assessment (HOMA) has been widely used in the clinical evaluation of insulin sensitivity, insulin resistance level, and islet β-cell function because of its simplicity and convenience. We calculated the following indicators: HOMA-IR= FPG*FINS/22.5, HOMA-β= 20×FINS/(FPG-3.5), and HOMA-ISI= 1/HOMA-IR (46). All subjects fasted for more than 10 hours overnight without drinking alcohol or engaging in vigorous exercise within 24 hours. Experienced professional nurses uniformly took venous blood from the antecubital vein using appropriate blood collection tubes in the early morning.

### Statistical analysis

Continuous variables are described as the means and standard deviations (SD). Statistical analyses were performed using SPSS version 26.0 (IBM Corp, Armonk, NY, USA). The paired *t-test* or analysis of variance (ANOVA) with Bonferroni’s test for comparisons was used to analyze the quantitative variables when the assumptions of normality and homogeneity of variance were satisfied; otherwise, the Mann–Whitney *U test* or the Kruskal–Wallis test was used. Differences between rates or ratios were determined using the χ2 test. Univariate and multivariate regressions were conducted to analyze the factors associated with DON presence in TAO patients. A P value < 0.05 was considered to be statistically significant.

## Results

### Demographics

A total of 146 participants were enrolled, with 98 TAO patients (57 developed DON and 28 bilateral DON patients) and 48 in the HC group. The demographic data of the patients are displayed in **Table 1**. There were no differences in sex distribution, height, weight, body mass index (BMI), or smoking history among the three groups. However, compared with the non-DON group, patients with DON had a higher mean age (50.38 ± 12.42 vs. 42.71 ± 13.25, P=0.037). The DON group had more patients with a BMI≥27 than the non-DON group (24.57% vs. 12.19%, P=0.077). The DON and total TAO groups had more hypertension than the HC group (P=0.027, P=0.048). T1DM was not found in our included patients. The total TAO group had higher numbers than the HC group for DM (26.53% vs 0, P<0.0001), hypertension (29.59% vs 14.58%, P=0.048), and hyperlipidemia (30.61% vs 4.17%, P<0.001). In the non-DON and DON group comparisons, no significant differences were found in the type of initial thyroid function, thyroid, or eye disease duration. The DON group had a higher frequency of steroid use (71.93% vs 51.22%, P=0.013); however, there was no significant statistical difference between them in the average steroid usage or time interval from the last steroid use to blood collection (P= 0.051, P= 0.817).

**Table 1:** Demographic characteristics of all enrolled subjects.

Variables	HC (n=48)	non-DON (n=41)	DON (n=57)	P1	P2	P3	P4
Sex, n (%) Male Female	21 (43.75) 27 (56.25)	21 (51.22) 20 (48.78)	31 (54.39) 26 (45.61)	0.482	0.278	0.757	0.290
Age, years	47.54 ± 17.05	42.71 ± 13.25	50.38 ± 12.42	0.361	0.724	0.037*	0.999
Height, cm Weight, kg	161.75 ± 9.16 63.41 ± 11.40	164.95 ± 6.92 62.73 ± 10.50	163.12 ± 8.89 65.27 ± 9.96	0.274 0.990	0.836 0.799	0.708 0.636	0.467 0.972
Body mass index, kg/m^2^ <18.5, n (%) 18.5-23.9, n (%) ≥24, n (%) ≥27, n (%)	24.08 ± 2.63 1 (2.08) 25 (52.08) 15 (31.25) 7 (14.59)	23.01 ± 3.23 3 (7.32) 20 (48.78) 13 (31.71) 5 (12.19)	24.50 ± 2.95 1 (1.75) 23 (40.35) 19 (33.33) 14 (24.57)	0.337	0.896	0.077	0.979
Smoking history, n (%)	13 (27.08)	13 (31.71)	23 (40.35)	0.633	0.154	0.381	0.246
Hypertension, n (%)	7 (14.58)	10 (24.39)	19 (33.33)	0.241	0.027*	0.339	0.048*
Diabetes mellitus, n (%)	0 (0.0)	11 (26.83)	15 (26.32)	**<0.0001** ****	**<0.0001** ****	0.955	**<0.0001** ****
Hyperlipidemia, n (%)	2 (4.17)	11 (26.83)	19 (33.33)	0.002**	**<0.001** ***	0.491	**<0.001** ***
Initial thyroid function Hyperthyroidism, n (%) Hypothyroidism, n (%) Euthyroidism, n (%)	–	33 (80.49) 6 (14.63) 2 (4.88)	50 (87.72) 5 (8.77) 2 (3.51)	–	–	0.611	–
Duration of TD, years	–	6.34 ± 4.32	5.34 ± 2.92	–	–	0.559	–
Duration of TAO, years	–	2.49 ± 2.11	2.08 ± 2.12	–	–	0.374	–
History of steroid use							
n (%)	–	21 (51.22)	43 (71.93)	–	–	0.013*	–
total dose (gram)	–	3.75 ± 3.02	3.87 ± 1.28	–	–	0.051	–
time interval (months)	–	4.85 ± 2.69	4.61 ± 2.98	–	–	0.817	–

HC, healthy control; DON, dysthyroid optic neuropathy; TD, thyroid dysfunction; TAO, Thyroid-associated ophthalmopathy; time interval: time interval from the last steroid use to blood collection; P1, HC versus non-DON; P2, HC versus DON; P3, non-DON versus DON; P4, HC versus total TAO; P values<0.05 are in bold.

*p < 0.05, **p < 0.01, ***p < 0.001, ****p<0.0001.

"-" represents not applicable.

### Ocular characteristics

A total of 88 healthy, 98 non-DON, and 78 DON eyes were included in the analysis (**Table 2**). Compared with the three groups, the DON group had significantly worse BCVA (P<0.0001). The exophthalmometry value was not significantly different between the non-DON and DON groups. Although IOP and CAS were quite different in the non-DON and DON comparisons, both IOPs were in the normal range, and the CAS mean values were below three points. Therefore, there were no clinical differences between the two groups.

**Table 2:** Clinical characteristics of all included eyes.

Variables	HC (n= 88)	non-DON (n= 98)	DON (n= 78)	P1	P2	P3	P4
BCVA, logMAR	0.00 ± 0.05	0.13 ± 0.23	0.70 ± 0.66	<0.0001****	<0.0001****	<0.0001****	<0.0001****
IOP, mmHg	15.86 ± 2.98	17.41 ± 3.78	19.60 ± 5.69	0.088	<0.0001****	0.125	<0.001***
Exophthalmometry value, mm	–	19.47 ± 3.69	19.71 ± 3.49	–	–	0.488	–
CAS	–	0.55 ± 0.75	1.33 ± 1.29	–	–	<0.0001****	–

BCVA, best-corrected visual acuity; IOP, intraocular pressure; CAS, clinical activity score.

P1, HC versus non-DON; P2, HC versus DON; P3, non-DON versus DON; P4, HC versus total TAO; P values<0.05 are in bold.

***p < 0.001, ****p<0.0001.

"-" represents not applicable.

### Laboratory measurements

**Table 3** compares the laboratory findings obtained from all the subjects. The TRAb level was higher in the DON group than in the non-DON group (P=0.043), while the TT3, TT4, FT3, FT4, TSH, TgAb, and TPOAb levels were not significantly different. The DON group had a higher FPG level than the HC and non-DON groups (P<0.05). The differences were more considerable with regard to the HbA1c level (both P<0.001). However, there were no significant differences between the three groups in FCP (P=1), FINS (P=1), HOMA-IR (P=1), HOMA-β (P>0.05), HOMA-ISI (P=1), or triglyceride (P>0.05) levels. Interestingly, the HOMA-IR level was highest in the DON groups than in the other two groups (HC 2.15 ± 0.89, non-DON 2.41 ± 1.24, and DON 2.82 ± 2.65), while the HOMA-β level was lowest (HC 101.8 ± 44.75%, non-DON 102.9 ± 54.61%, and DON 88.29 ± 52.75%). One patient in each of the NC and DON groups was GADA-positive, while IAA, ICA, IA-2A, and ZnT8A positivity was not found. In addition, the DON group seemed to have a higher total serum cholesterol level than the other two groups (P=0.002, 0.052).

**Table 3:** Laboratory characteristics of all enrolled subjects.

Variables	HC (n= 48)	non-DON (n= 41)	DON (n= 57)	P1	P2	P3	P4
TT3 (1.3-3.1 nmol/l)	–	1.89 ± 0.69	2.01 ± 0.71	–	–	0.079	–
TT4 (66-181 nmol/l)	–	106.45 ± 27.73	106.94 ± 32.21	–	–	0.925	–
FT3 (3.1-6.8 pmol/l)	–	5.21 ± 1.06	5.57 ± 3.17	–	–	0.920	–
FT4 (12-22 pmol/l)	–	17.16 ± 4.07	17.42 ± 6.69	–	–	0.849	–
TSH (0.27-4.2 μIU/ml)	–	2.41 ± 3.56	3.13 ± 5.71	–	–	0.589	–
TgAb (0-30 IU/ml)	–	179.77 ± 513.66	236.32 ± 653.23	–	–	0.384	–
TPOAb (≤34.0 IU/ml)	–	68.94 ± 139.23	67.30 ± 125.67	–	–	0.349	–
TRAb (≤1.75 IU/l)	–	8.27 ± 16.52	27.04 ± 61.69	–	–	0.043*	–
FPG (4.1-5.9 mmol/l)	5.35 ± 0.39	5.51 ± 0.68	6.05 ± 1.15	1	<0.001***	0.016*	0.019*
HbA1c (3-6%)	5.48 ± 0.75	5.79 ± 0.49	6.47 ± 1.02	0.089	<0.0001****	<0.001***	<0.0001****
FCP (0.26-1.39 nmol/l)	0.62 ± 0.15	0.64 ± 0.20	0.66 ± 0.28	1	1	1	1
FINS (3.08-15.59 μU/mL)	9.01 ± 3.53	9.69 ± 4.59	10.23 ± 7.47	1	1	1	1
HOMA-IR	2.15 ± 0.89	2.41 ± 1.24	**2.82 ± 2.65**	1	1	1	1
HOMA-ISI	0.57 ± 0.33	0.64 ± 0.69	0.76 ± 2.02	1	1	1	1
HOMA-β (%)	101.8 ± 44.75	102.9 ± 54.61	**88.29 ± 52.75**	1	0.429	0.906	1
Antibodies IAA, n (%) ICA, n (%) GADA, n (%) IA-2A, n (%) ZnT8A, n (%)	0 0 1 (2.08) 0 0	0 0 0 0 0	0 0 1 (1.75) 0 0	–	–	–	–
total serum cholesterol (<5.2 mmol/l)	4.93 ± 0.76	5.08 ± 0.83	5.52 ± 0.86	0.824	0.002**	0.052	0.029*
triglycerides (<1.69 mmol/l)	0.77 ± 0.34	0.96 ± 1.11	1.04 ± 0.66	1	0.471	0.131	1

Data indicate mean values ± SD.

P1, HC versus non-DON; P2, HC versus DON; P3, non-DON versus DON; P4, HC versus total TAO; P values<0.05 are in bold.

*p < 0.05, **p < 0.01, ***p < 0.001, ****p< 0.0001.

"-" represents not applicable.

Then, to clarify whether these diabetes-related indicator differences only existed in DM patients or were also applicable to non-DM patients, we conducted a statistical analysis for patients with and without DM, respectively (**Table 4**). The results showed that the FPG, FCP, and FINS levels in non-DM patients were all within the normal reference range. The HbA1c level of the DON group was significantly higher than that of the non-DON group in both non-DM patients (P=0.020) and DM patients (P<0.0001). The average time interval of the two groups exceeded 3 months, and there was no statistical difference between the two groups (P= 0.817). HbA1c reflects the average blood glucose level 2 to 3 months before the test and is unaffected by diet or exercise. Therefore, steroid usage history may have little effect on HbA1c in our current study. The DON with DM group had a higher FPG level than the non-DON with DM group (P=0.023). DON with DM patients showed the highest HOMA-IR and the lowest HOMA- β, which indicated that individual insulin resistance increased and islet β cell function decreased.

**Table 4:** Laboratory characteristics of TAO subjects.

(A) non-DON with non-DM (n=30)		(B) non-DON with DM (n=11)	(C) DON with non-DM (n=42)	(D) DON with DM (n=15)	Group A vs C P value	Group B vs D P value
HbA1c (3-6%)	5.64 ± 0.35	6.21 ± 0.60	6.07 ± 0.41	7.59 ± 1.37	0.020*	<0.0001****
FPG (4.1-5.9mmol/l)	5.31 ± 0.44	6.07 ± 0.91	5.69 ± 0.49	7.03 ± 1.77	0.120	0.023*
FCP (0.26-1.39nmol/l)	0.62 ± 0.19	0.69 ± 0.22	0.63 ± 0.18	0.76 ± 0.45	1	1
FINS (3.08-15.59 μU/mL)	9.37 ± 4.45	10.56 ± 5.07	9.24 ± 4.03	13.02 ± 12.81	1	1
HOMA-IR	2.22 ± 1.23	2.89 ± 1.47	2.33 ± 1.02	**4.18 ± 4.73**	1	1
HOMA-ISI	0.68 ± 0.77	0.51 ± 0.44	0.53 ± 0.27	1.43 ± 3.94	1	1
HOMA-β (%)	108.70 ± 56.00	87.16 ± 49.65	89.79 ± 46.80	**84.09 ± 68.50**	0.918	1

Data indicate mean values ± SD. P-values<0.05 are in bold.

*p < 0.05, ****p<0.0001.

### Factors associated with DON presence in TAO patients

To identify factors associated with DON presence in TAO patients, we performed a multivariate analysis using only those significant factors in univariate analysis (**Table 5**). On univariate analysis, age (P=0.006), BMI (P=0.022), history of steroid use (P=0.014), levels of FPG (P=0.013), HbA1c (P=0.001), and total serum cholesterol (P=0.016) were significantly associated with the presence/absence of DON. After adjusting for other significant factors in multivariate analysis, the HbA1c level was an independent factor related to DON presence in TAO patients, with P=0.009 and odds ratio (OR)=4.012 [95% confidence interval=1.414-11.382].

**Table 5:** Logistic regression analysis with the presence of DON as the dependent variable.

Independent variables	Univariate regression analysis		Multivariate regression analysis	
	OR (95% CI)	P-value	OR (95% CI)	P-value
Age	**1.048 (1.013-1.084)**	0.006**	1.021 (0.979-1.064)	0.337
Body mass index	**1.172 (1.023-1.342)**	0.022*	1.120 (0.948-1.323)	0.183
Smoking history	1.457 (0.626-3.389)	0.382	–	–
Hypertension	1.550 (0.630-3.815)	0.340	–	–
Diabetes mellitus	0.974 (0.393-2.415)	0.955	–	–
Hyperlipidemia	1.364 (0.564-3.299)	0.491	–	–
History of steroid use	**2.925 (1.238-6.909)**	0.014*	2.516 (0.905-6.990)	0.077
FPG	**2.259 (1.185-4.305)**	0.013*	1.332 (0.572-3.105)	0.506
HbA1c	**5.819 (2.132-15.880)**	0.001***	**4.012 (1.414-11.382)**	0.009**
FCP	1.567 (0.295-8.308)	0.598	–	–
FINS	1.014 (0.949-1.083)	0.679	–	–
HOMA-IR	1.115 (0.876-1.420)	0.377	–	–
HOMA-ISI	1.058 (0.793-1.411)	0.700	–	–
HOMA-β	0.995 (0.987-1.002)	0.187	–	–
total serum cholesterol	**1.889 (1.129-3.162)**	0.016*	1.847 (0.979-3.486)	0.058
triglycerides	1.115 (0.687-1.811)	0.659	–	–

OR, Odds ratio; 95%CI, 95% confidence interval. P values<0.05 are in bold.

*p < 0.05, **p < 0.01, ***p < 0.001.

"-" represents not applicable.

## Discussion

The exact pathogenesis of DON remains unclear but might be related to the mechanical compression.

from the enlarged extraocular muscles and excessive soft tissue (47). Among the various theories, the leading hypothesis at present is that the optic nerve is compressed directly by enlarged extraocular muscles (EOMs) located at the orbital apex. In this study, we present data on the serum levels of selected diabetes-related indicators and their changes associated with TAO and DON. Previous studies have shown that DM is a risk factor for TAO (27, 28, 42). The prevalence of T1DM in patients with TAO is higher than that in healthy individuals (42). TAO patients with DM had more DON and had a worse visual prognosis than the whole TAO group (42). Furthermore, TAO can be more frequent and severe in T2DM patients and is significantly related to obesity and is the course of diabetes and diabetic vasculopathy (28). Treatment of T2DM can affect the onset and progression of TAO, including thiazolidinediones (48) and biguanides (49).

Islet autoantibodies exist in 6.4% of T2DM overweight/obese adults, including those who are severely obese and have unique clinical characteristics, and islet autoantibodies play a role in predicting insulin demand for T2DM (50). In our study, only one case of GADA positivity was found in each of the HC and DON groups. No positive IAA, ICA, IA-2A, or ZnT8A expression was found. However, in previous studies, it was found that patients with AITD (866 patients), especially those with Graves’ disease, are prone to develop β-cell autoimmunity and insulin-requiring diabetes, particularly those with a high titer of GADA and/or who are positive for both GADA and IA-2A (51). In 92 cases of AITD, 15% of patients were found to produce specific IAA (52). This difference may be due to the different compositions of thyroid dysfunction in our included study subjects.

IR is a common risk factor for various pathophysiological states and diseases, such as T2DM, hypertension, metabolic syndrome, cardiovascular disease, and lipid metabolism disorders. T2DM and thyroid dysfunction are both dysfunctional metabolic diseases, and their combination makes the condition more complicated and increases the risk of cardiovascular disease, which makes clinical treatment difficult. HOMA gives an estimate of basal insulin resistance (HOMA-IR, used to evaluate individual insulin resistance levels; HOMA-ISI, used to evaluate individual insulin sensitivity; HOMA- β, used to evaluate individual islets β indicators of cell function) (53). Decreased islet β-cell function was observed, and 34.7% had persistent abnormalities during follow-up (54). In our study, we did not observe any significant differences in HOMA-IR, HOMA-β, or HOMA-ISI. However, DON with DM patients showed the highest HOMA-IR and the lowest HOMA-β. This suggests that DON with DM patients’ IR increased and islet β cell function decreased. In addition, the decrease in HOMA- β was also observed in DON with non-DM patients. Whether DON patients have a decrease in early islet β cell function or IR needs further study. HOMA indicators deserve to be paid attention to in a large sample population.

We noted substantial differences in FPG and HbA1c levels between the HC and TAO groups, as well as in the non-DON and DON groups. The HbA1c level of the DON group was significantly higher than that of the non-DON group in both non-DM and DM patients. The DON with DM group had a higher FPG level than the non-DON with DM group. Similar to a study of 40 hyperthyroid patients, FPG and HOMA-IR tended to be higher than euthyroidism, indicating the existence of IR in hyperthyroidism (55). Abnormal glucose tolerance is a critical metabolic outcome in patients with Graves’ disease. A previous study showed that baseline HbA1c levels were found to be significantly higher in hypothyroid patients (56). In our included objects, we did not find that the HbA1c of hypothyroidism patients was higher than that of non-hypothyroidism patients, which may be related to the small number of people we included (data not shown). Moreover, most of the TAO patients treated in our hospital have already undergone thyroid function adjustment in the endocrine department, which may affect the level of HbA1c. HbA1c levels were decreased by thyroid hormone replacement (57). On univariate analysis, age, BMI, history of steroid use, and levels of FPG, HbA1c, and total serum cholesterol were significantly associated with the presence/absence of DON. Intriguingly, in a multivariate analysis, HbA1c was significantly associated with the presence/absence of DON after adjusting for clinical factors. In addition, HbA1c levels are dependent not only on the average blood glucose levels over the preceding 2- 3 months but also on the turnover of erythrocytes (58). Disorders characterized by altered erythrocyte turnover can cause HbA1c perturbations independent of the glycemic status by changing the relative percentages of young and old erythrocytes in peripheral blood, thereby affecting the mean age of the RBCs in circulation (59). Regarding hyperthyroidism, a faster turnover would be anticipated to decrease the average age of red blood cells in circulation, resulting in an erroneous decrease of HbA1c in relation to the level of glycemia. Therefore, the use of HbA1c as a potential biomarker for DON warrants further investigation.

Some limitations in this study need to be noted. First, our study was a cross-sectional study. TAO patients were not followed up to assess whether there was an association between blood glucose and lipid-related indicators and changes in eye disease. Second, we did not perform the appropriate tests for abnormal glucose tolerance (i.e., OGTT). The levels of HbA1c, FPG, and the frequency of IR in each group need to be further explored by expanding the sample size. Third, we included only patients who visited or were referred to a single university hospital, which may have introduced selection bias.

In conclusion, age, BMI, and levels of FPG, HbA1c, and CHOL were closely related to DON. HbA1c is an independent risk factor for DON. Islet β-cell autoantibodies and IR were not found to be significantly different between the HC and TAO groups. However, the level of HOMA-IR and HOMA-β was abnormal in DON patients. Coexisting abnormal HbA1c may predict the worsening of visual function in TAO patients. Given the interconnected link between thyroid dysfunction and DM, the use of HbA1c as a potential biomarker for DON warrants further investigation. In short, to a certain extent, thyroid dysfunction/TAO and DM are interdependent and influence each other in the occurrence and development of diseases, which play a crucial role in patients’ quality of life.

## Data availability statement

The datasets generated during and analyzed during the current study are not publicly available but are available from the corresponding author on reasonable request.

## Ethics statement

The studies involving humans were approved by The Institutional Review Board of Zhongshan Ophthalmic Center (2016KYPJ028). The studies were conducted in accordance with the local legislation and institutional requirements. The participants provided their written informed consent to participate in this study.

## Author contributions

Data curation, XW; Formal analysis, HJY and RXC; Investigation, XW; Methodology, SLY and TZ; Project administration, HSY; Software, HJY and SLY; Supervision, HSY; Validation, WX; Writing – original draft, XW; Writing – review and editing, HJY, RXC, and HSY. All authors contributed to the article and approved the submitted version.

## References

[1] Smith T Hegedüs L. Graves’ Disease. New Engl J Med (2016) 375(16):1552–65. doi: 10.1056/NEJMra1510030 27797318

[2] Bahn R. Graves’ ophthalmopathy. New Engl J Med (2010) 362(8):726–38. doi: 10.1056/NEJMra0905750 PMC3902010 20181974

[3] Taylor PN Zhang L Lee RWJ Muller I Ezra DG Dayan CM. New insights into the pathogenesis and nonsurgical management of Graves orbitopathy. Nat Rev Endocrinol (2020) 16(2):104–16. doi: 10.1038/s41574-019-0305-4 31889140

[4] Wiersinga W Bartalena L. Epidemiology and prevention of Graves’ ophthalmopathy. Thyroid (2002) 12(10):855–60. doi: 10.1089/105072502761016476 12487767

[5] Stan M Bahn R. Risk factors for development or deterioration of Graves’ ophthalmopathy. Thyroid (2010) 20(7):777–83. doi: 10.1089/thy.2010.1634 PMC3357079 20578901

[6] Eckstein AK Plicht M Lax H Hirche H Quadbeck B Mann K. Clinical results of anti-inflammatory therapy in Graves’ ophthalmopathy and association with thyroidal autoantibodies. Clin Endocrinol (Oxf) (2004) 61(5):612–8. doi: 10.1111/j.1365-2265.2004.02143.x 15521965

[7] Jang SY Shin DY Lee EJ Choi YJ Lee SY Yoon JS. Correlation between TSH receptor antibody assays and clinical manifestations of Graves’ orbitopathy. Yonsei Med J (2013) 54(4):1033–9. doi: 10.3349/ymj.2013.54.4.1033 PMC3663223 23709442

[8] Saeedi P Petersohn I Salpea P Malanda B Karuranga S Unwin N. Global and regional diabetes prevalence estimates for 2019 and projections for 2030 and 2045: Results from the International Diabetes Federation Diabetes Atlas, 9(th) edition. Diabetes Res Clin Pract (2019) 157:107843. doi: 10.1016/j.diabres.2019.107843 31518657

[9] Luo Z Fabre G Rodwin VG. Meeting the challenge of diabetes in China. Int J Health Policy Manage (2020) 9(2):47–52. doi: 10.15171/ijhpm.2019.80 PMC7054646 32124588

[10] Shun C Donaghue K Phelan H Twigg S Craig M. Thyroid autoimmunity in Type 1 diabetes: systematic review and meta-analysis. Diabetic medicine: J Br Diabetic Assoc (2014) 31(2):126–35. doi: 10.1111/dme.12318 24103027

[11] Umpierrez G Latif K Murphy M Lambeth H Stentz F Bush A. Thyroid dysfunction in patients with type 1 diabetes: a longitudinal study. Diabetes Care (2003) 26(4):1181–5. doi: 10.2337/diacare.26.4.1181 12663594

[12] Eisenbarth GS Gottlieb PA. Autoimmune polyendocrine syndromes. N Engl J Med (2004) 350(20):2068–79. doi: 10.1056/NEJMra030158 15141045

[13] Tomer Y Menconi F. Type 1 diabetes and autoimmune thyroiditis: the genetic connection. Thyroid (2009) 19(2):99–102. doi: 10.1089/thy.2008.1565 19191741 PMC2729687

[14] Wilkin T Hammonds P Mirza I Bone A Webster K. Graves’ disease of the beta cell: glucose dysregulation due to islet-cell stimulating antibodies. Lancet (London England) (1988) 2(8621):1155–8. doi: 10.1016/S0140-6736(88)90232-2 2903375

[15] Gu Y Li H Bao X Zhang Q Liu L Meng G. The relationship between thyroid function and the prevalence of type 2 diabetes mellitus in euthyroid subjects. J Clin Endocrinol Metab (2017) 102(2):434–42. doi: 10.1210/jc.2016-2965 27906594

[16] Jun J Jee J Bae J Jin S Hur K Lee M. Association between changes in thyroid hormones and incident type 2 diabetes: A seven-year longitudinal study. Thyroid (2017) 27(1):29–38. doi: 10.1089/thy.2016.0171 27809684

[17] Biondi B Kahaly G Robertson R. Thyroid dysfunction and diabetes mellitus: two closely associated disorders. Endoc Rev (2019) 40(3):789–824. doi: 10.1210/er.2018-00163 PMC6507635 30649221

[18] Pihoker C Gilliam L Hampe C Lernmark A. Autoantibodies in diabetes. Diabetes (2005) 54(Suppl 2):S52–S61. doi: 10.2337/diabetes.54.suppl_2.S52 16306341

[19] Ziegler A Rewers M Simell O Simell T Lempainen J Steck A. Seroconversion to multiple islet autoantibodies and risk of progression to diabetes in children. JAMA (2013) 309(23):2473–9. doi: 10.1001/jama.2013.6285 PMC4878912 23780460

[20] Yamaguchi Y Chikuba N Ueda Y Yamamoto H Yamasaki H Nakanishi T. Islet cell antibodies in patients with autoimmune thyroid disease. Diabetes (1991) 40(3):319–22. doi: 10.2337/diab.40.3.319 1999272

[21] Kawasaki E Takino H Yano M Uotani S Matsumoto K Takao Y. Autoantibodies to glutamic acid decarboxylase in patients with IDDM and autoimmune thyroid disease. Diabetes (1994) 43(1):80–6. doi: 10.2337/diab.43.1.80 8262321

[22] Maugendre D Verite F Guilhem I Genetet B Allannic H Delamaire M. Anti-pancreatic autoimmunity and Graves’ disease: study of a cohort of 600 Caucasian patients. Eur J Endocrinol (1997) 137(5):503–10. doi: 10.1530/eje.0.1370503 9405030

[23] Khassawneh AH Al-Mistarehi AH Zein Alaabdin AM Khasawneh L AlQuran TM Kheirallah KA. Prevalence and predictors of thyroid dysfunction among type 2 diabetic patients: A case-control study. Int J Gen Med (2020) 13:803–16. doi: 10.2147/IJGM.S273900 PMC7568427 33116772

[24] Vamshidhar IS Rani SSS. A study of association of thyroid dysfunctions in patients with type 2 diabetes mellitus. Maedica (Bucur) (2020) 15(2):169–73. doi: 10.26574/maedica.2020.15.2.169 PMC7482697 32952680

[25] Gray RS Herd R Clarke BF. The clinical features of diabetes with coexisting autoimmune thyroid disease. Diabetologia (1981) 20(6):602–6. doi: 10.1007/BF00257427 7262475

[26] Roti E Braverman LE Robuschi G Salvi M Gardini E d’Amato L. Basal and glucose- and arginine-stimulated serum concentrations of insulin, C-peptide, and glucagon in hyperthyroid patients. Metabolism (1986) 35(4):337–42. doi: 10.1016/0026-0495(86)90151-4 3515118

[27] Rath S Pattnaik M Tripathy D Mohapatra S Panigrahy B Ali MH. Sight-threatening thyroid eye disease: role of diabetes mellitus and interaction with other risk factors. Ophthalmic Plast Reconstr Surg (2021) 37(4):352–60. doi: 10.1097/IOP.0000000000001871 33060509

[28] Le Moli R Muscia V Tumminia A Frittitta L Buscema M Palermo F. Type 2 diabetic patients with Graves’ disease have more frequent and severe Graves’ orbitopathy. Nutrition metabolism Cardiovasc diseases: NMCD (2015) 25(5):452–7. doi: 10.1016/j.numecd.2015.01.003 25746910

[29] Ramamurthy LB Rangarajan V Srirao N Malini B Bansal R Yuvarajan K. Severity of thyroid eye disease and type-2 diabetes mellitus: Is there a correlation? Indian J Ophthalmol (2020) 68(6):1127–31. doi: 10.4103/ijo.IJO_1443_19 PMC7508111 32461446

[30] Tack C Stienstra R Joosten L Netea M. Inflammation links excess fat to insulin resistance: the role of the interleukin-1 family. Immunol Rev (2012) 249(1):239–52. doi: 10.1111/j.1600-065X.2012.01145.x 22889226

[31] Hiromatsu Y Yang D Bednarczuk T Miyake I Nonaka K Inoue Y. Cytokine profiles in eye muscle tissue and orbital fat tissue from patients with thyroid-associated ophthalmopathy. J Clin Endocrinol Metab (2000) 85(3):1194–9. doi: 10.1210/jcem.85.3.6433 10720061

[32] Gianoukakis AG Khadavi N Smith TJ. Cytokines, Graves’ disease, and thyroid-associated ophthalmopathy. Thyroid (2008) 18(9):953–8. doi: 10.1089/thy.2007.0405 PMC2879490 18713026

[33] Mottillo E Desjardins E Crane J Smith B Green A Ducommun S. Lack of adipocyte AMPK exacerbates insulin resistance and hepatic steatosis through brown and beige adipose tissue function. Cell Metab (2016) 24(1):118–29. doi: 10.1016/j.cmet.2016.06.006 PMC5239668 27411013

[34] Krieger CC Neumann S Gershengorn MC. TSH/IGF1 receptor crosstalk: Mechanism and clinical implications. Pharmacol Ther (2020) 209:107502. doi: 10.1016/j.pharmthera.2020.107502 32061922 PMC7187798

[35] Smith TJ Janssen J. Insulin-like growth factor-I receptor and thyroid-associated ophthalmopathy. Endocr Rev (2019) 40(1):236–67. doi: 10.1210/er.2018-00066 PMC6338478 30215690

[36] Greenhill C. IGFs potential biomarkers for type 1 diabetes mellitus. Nat Rev Endocrinol (2020) 16(3):130–1. doi: 10.1038/s41574-020-0317-0 31907399

[37] Ranke MB. Insulin-like growth factor-I treatment of growth disorders, diabetes mellitus and insulin resistance. Trends Endocrinol Metab (2005) 16(4):190–7. doi: 10.1016/j.tem.2005.03.011 15860416

[38] Mehran L Amouzegar A Tohidi M Moayedi M Azizi F. Serum free thyroxine concentration is associated with metabolic syndrome in euthyroid subjects. Thyroid (2014) 24(11):1566–74. doi: 10.1089/thy.2014.0103 25069017

[39] Wen S Jiang W Zhou L. Islet autoantibodies in the patients with sjogren’s syndrome and thyroid disease and risk of progression to latent autoimmune diabetes in adults: A case series. Diabetes Metab Syndr Obes (2021) 14:1025–33. doi: 10.2147/DMSO.S295847 PMC7943329 33707961

[40] Aksoy DY Yurekli BP Yildiz BO Gedik O. Prevalence of glutamic acid decarboxylase antibody positivity and its association with insulin secretion and sensitivity in autoimmune thyroid disease: A pilot study. Exp Clin Endocrinol Diabetes (2006) 114(8):412–6. doi: 10.1055/s-2006-924153 17039421

[41] Li Q Lu M Wang NJ Chen Y Chen YC Han B. Relationship between free thyroxine and islet beta-cell function in euthyroid subjects. Curr Med Sci (2020) 40(1):69–77. doi: 10.1007/s11596-020-2148-6 32166667

[42] Kalmann R Mourits M. Diabetes mellitus: a risk factor in patients with Graves’ orbitopathy. Br J ophthalmol (1999) 83(4):463–5. doi: 10.1136/bjo.83.4.463 PMC1723016 10434871

[43] Bartley G Gorman C. Diagnostic criteria for Graves’ ophthalmopathy. Am J ophthalmol (1995) 119(6):792–5. doi: 10.1016/S0002-9394(14)72787-4 7785696

[44] Bartalena L Baldeschi L Boboridis K Eckstein A Kahaly G Marcocci C. The 2016 european thyroid association/european group on graves’ Orbitopathy guidelines for the management of graves’ Orbitopathy. Eur Thyroid J (2016) 5(1):9–26. doi: 10.1159/000443828 27099835 PMC4836120

[45] Dolman PJ. Dysthyroid optic neuropathy: evaluation and management. J Endocrinol Invest (2021) 44(3):421–9. doi: 10.1007/s40618-020-01361-y 32729049

[46] Matthews DR Hosker JP Rudenski AS Naylor BA Treacher DF Turner RC. Homeostasis model assessment: insulin resistance and beta-cell function from fasting plasma glucose and insulin concentrations in man. Diabetologia (1985) 28(7):412–9. doi: 10.1007/BF00280883 3899825

[47] Zhang T Xiao W Ye H Chen R Mao Y Yang H. Peripapillary and macular vessel density in dysthyroid optic neuropathy: an optical coherence tomography angiography study. Invest Ophthalmol Vis Sci (2019) 60(6):1863–9. doi: 10.1167/iovs.18-25941 31042792

[48] Urselli F Pontieri G Peschi L Liccardi A Ruggiero A Biondi B. Active moderate-to-severe graves’ Orbitopathy in a patient with type 2 diabetes mellitus and vascular complications. Front endocrinol (2018) 9:810. doi: 10.3389/fendo.2018.00810 PMC6344418 30705666

[49] Han Y Hwang S Kim J Byun J Yoon J Lee E. Biguanides metformin and phenformin generate therapeutic effects via AMP-activated protein kinase/extracellular-regulated kinase pathways in an *in vitro* model of graves’ Orbitopathy. Thyroid (2018) 28(4):528–36. doi: 10.1089/thy.2017.0338 29589999

[50] Pilla SJ Balasubramanyam A Knowler WC Lazo M Nathan DM Pi-Sunyer X. Islet autoantibody positivity in overweight and obese adults with type 2 diabetes. Autoimmunity (2018) 51(8):408–16. doi: 10.1080/08916934.2018.1547711 PMC6362362 30661481

[51] Moriguchi M Noso S Kawabata Y Yamauchi T Harada T Komaki K. Clinical and genetic characteristics of patients with autoimmune thyroid disease with anti-islet autoimmunity. Metabolism (2011) 60(6):761–6. doi: 10.1016/j.metabol.2010.07.025 20825955

[52] Vardi P Modan-Mozes D Ish-Shalom S Soloveitzik L Barzilai D Modan M. Low titer, competitive insulin autoantibodies are spontaneously produced in autoimmune diseases of the thyroid. Diabetes Res Clin Pract (1993) 21(2-3):161–6. doi: 10.1016/0168-8227(93)90064-C 8269817

[53] Wallace TM Matthews DR. The assessment of insulin resistance in man. Diabetes Med (2002) 19(7):527–34. doi: 10.1046/j.1464-5491.2002.00745.x 12099954

[54] Lakshmana Perumal N Selvi J Sridharan K Sahoo J Kamalanathan S. Insulin sensitivity and beta-cell function in graves’ Disease and their changes with the carbimazole-induced euthyroid state. Eur Thyroid J (2019) 8(2):59–63. doi: 10.1159/000496924 31192143 PMC6514524

[55] Agbaht K Erdogan MF Emral R Baskal N Gullu S. Circulating glucagon to ghrelin ratio as a determinant of insulin resistance in hyperthyroidism. Endocrine (2014) 45(1):106–13. doi: 10.1007/s12020-013-9951-9 23572405

[56] Bhattacharjee R Thukral A Chakraborty PP Roy A Goswami S Ghosh S. Effects of thyroid status on glycated hemoglobin. Indian J Endocrinol Metab (2017) 21(1):26–30. doi: 10.4103/2230-8210.196017 28217494 PMC5240076

[57] Kim MK Kwon HS Baek KH Lee JH Park WC Sohn HS. Effects of thyroid hormone on A1C and glycated albumin levels in nondiabetic subjects with overt hypothyroidism. Diabetes Care (2010) 33(12):2546–8. doi: 10.2337/dc10-0988 PMC2992186 20823345

[58] Mani Deepthi D Vaikkakara S Patil A Ganta S Sachan A Raghavendra K. Effect of correction of hyperthyroidism with anti-thyroid drugs on the glycated hemoglobin in non-diabetic patients with primary hyperthyroidism. Int J Endocrinol Metab (2021) 19(1):e105751. doi: 10.5812/ijem.105751 33815517 PMC8010563

[59] Fitzgibbons JF Koler RD Jones RT. Red cell age-related changes of hemoglobins AIa+b and AIc in normal and diabetic subjects. J Clin Invest (1976) 58(4):820–4. doi: 10.1172/JCI108534 PMC333244 965489

